# Increased Prevalence of Minor Physical Anomalies Among the Healthy First-Degree Relatives of Bipolar I Patients – Results With the Méhes Scale

**DOI:** 10.3389/fpsyt.2021.672241

**Published:** 2021-04-29

**Authors:** Tímea Csulak, Györgyi Csábi, Róbert Herold, Viktor Vörös, Sára Jeges, András Hajnal, Márton Áron Kovács, Maria Simon, Márton Herold, Ákos Levente Tóth, Tamás Tényi

**Affiliations:** ^1^Department of Psychiatry and Psychotherapy, Medical School, University of Pécs, Pécs, Hungary; ^2^Department of Pediatrics, Medical School, University of Pécs, Pécs, Hungary; ^3^Faculty of Health Sciences, Institute of Nursing and Patients Care, University of Pécs, Pécs, Hungary; ^4^Faculty of Sciences, Institute of Physical Education and Sport Science, University of Pécs, Pécs, Hungary

**Keywords:** psychotic disorders, somatic markers, endophenotype, bipolar disorder, neurodevelopment

## Abstract

Minor physical anomalies are somatic markers of aberrant neurodevelopment, so the higher prevalence of these signs among the relatives of bipolar I patients can confirm minor physical anomalies as endophenotypes. The aim of the study was to evaluate the prevalence of minor physical anomalies in first-degree healthy relatives of patients with bipolar I disorder compared to normal control subjects. Using a list of 57 minor physical anomalies (the Méhes Scale), 20 first-degree unaffected relatives of patients with the diagnosis of bipolar I disorder and as a comparison 20 matched normal control subjects were examined. Minor physical anomalies were more common in the ear, head, mouth and trunk regions among the relatives of bipolar I patients compared to normal controls. By the differentiation of minor malformations and phenogenetic variants, we have found that both minor malformations and phenogenetic variants were more common among the relatives of bipolar I patients compared to the control group, while individual analyses showed, that one minor malformation (sole crease) and one phenogenetic variant (high arched palate) were more prevalent in the relative group. This is the first report in literature on the increased prevalence of minor physical anomalies among the first-degree unaffected relatives of bipolar I patients. The study support the concept, that minor physical anomalies can be endophenotypic markers of bipolar I affective disorder.

## Introduction

Minor physical anomalies (MPAs) are clinically nonsignificant errors of morphogenesis which have a prenatal origin and may bear important informational value. The presence of minor physical anomalies is a sensitive indicator of altered embryonic development. Since both the brain and the skin derived from the ectoderm, minor physical anomalies may be physical markers of aberrant neurodevelopment. Minor physical anomalies develop during the first and/or early second trimester of gestation (1–5) and represent potentially valuable indices of disturbances in early brain development. “Once formed they persist into adult life and can be detected on visual examination of the particular body area” (9, 224.pp.). As our group (4, 6–10) and others (11, 12) have discussed earlier, differences and contradictions between studies on minor physical anomalies among adults and children with different neuropsychiatric disorders, may be associated, partly, with the problems in the use of the Waldrop-scale for the detection of these signs. The Waldrop-scale contains only 18 minor physical anomalies (13) while in recent genetic and pediatric literature more than 50 anomalies have been listed (1, 2, 8, 12). A major problem with the Waldrop-scale that it makes no distinction between minor malformations, which arise during organogenesis and phenogenetic variants, which appear after organogenesis (1, 2, 7). A clear distinction between morphogenetic events developing during and after organogenesis seems to be needed. Minor malformations are always abnormal and are qualitive defects of embryogenesis, which arise during organogenesis. All malformations are developmental field defects and usually they are all-or-none anomalies. In contrast phenogenetic variants are quantitative defects of final morphogenesis and arise after organogenesis” (9,224.pp.). Using a list of MPAs containing 57 minor signs evaluated by Méhes (2), previously we have studied the prevalence of minor physical anomalies in patients with schizophrenia, bipolar affective disorder, alcohol dependence, Tourette syndrome, major depression and among the healthy relatives of patients with schizophrenia (3, 6, 8–10, 14–16), and the list and definitions has become also acceptable for researchers (8).

The endophenotype concept of bipolar affective disorder represents an important approach in the exploration of the pathogenesis of the illness. Gottesman and Gould (17) described an endophenotype as an intermediate phenotype that fills the gap between genes and diseases. “Endophenotypes should be: (1) associated with the illness, (2) heritable, (3) state-independent, (4) found in unaffected relatives at a higher rate than in the general population, and (5) shown to co-segregate with the illness within families” (9,225.pp.). An important characteristic of an endophenotype, that it can be more commonly found among the healthy, first-degree relatives of patients with schizophrenia (for a review in schizophrenia, see 9). Minor physical anomalies are suggested as endophenotypes on account of the findings that in some (5, 10–12, 18, 19), but not all (20–22) studies MPAs were more common in bipolar patients than in healthy controls, MPAs are trait-markers, so they can be detected in remission too.

Only one single study (22) was reported with a very low number of participants (9 siblings of bipolar patients) on the prevalence of minor anomalies in bipolar relatives, where no difference was detected among the healthy first-degree relatives compared to normal controls, so we consider the field to be open for further research.

The aim of this study was to investigate the prevalence of minor physical anomalies - using the Méhes Scale to differentiate minor malformations and phenogenetic variants - in the relatives of patients with bipolar I disorder comparing them to normal control subjects. The following hypotheses have been tested: (1) MPAs are more prevalent in the relatives of bipolar I patients compared to normal controls, which supports the hypothesis, that MPAs can be endophenotypic markers of bipolar I disorder (2) a higher rate of MPAs is found predominantly in the craniofacial regions among the relatives of bipolar I patients, pointing at aberrant early (first and second trimester) neurodevelopment.

## Materials and Methods

### Study Subjects

Using a list of 57 minor physical anomalies collected by Méhes (2), 20 first-degree healthy relatives of patients with the diagnosis of bipolar I disorder were examined. Eleven parents, 3 siblings and 6 children were included in the study, the mean age of the relatives was 57.20 years (standard deviation: 21.53, standard error mean: 4.81). The age of the 6 children were: 44, 47, 35, 42, 42 and 28 years. The sample size followed previous publication (9). As a comparison 20 normal control subjects matched for sex, age and ethnic origin were also observed for minor physical anomalies. Controls were excluded if they endorsed any personal or family history (in the first- or second-degree relatives) of psychotic disorders, mood disorders, personality disorders and anxiety disorders. First-degree relatives of bipolar I patients were excluded if they endorsed a personal history of psychotic disorders, mood disorders, personality disorders and anxiety disorders. For all participants, psychotic disorders, mood disorders, personality disorders and anxiety disorders were ruled out independently by two experienced psychiatrists according to the DSM-5 (23). All clinical information were obtained from structured clinical interviews.

### Examination of Minor Physical Anomalies

We have used the Méhes Scale for evaluation of MPAs, which includes 57 minor signs (4, 6, 8–10). MPAs were connected to body regions for comparison. A clear differentiation between minor malformations and phenogenetic variants were introduced, the scale and detailed definitions were published earlier (8). All participants gave informed consent, the study was performed in accordance with the Declaration of Helsinki and was evaluated following institutional guidelines. The Commitee on Medical Ethics of University of Pécs accepted the proposal for the study (No.6416). Two examiners (T. Tényi, Gy. Csábi) investigated all the relatives and controls separately. “The raters were trained by Professor Károly Méhes, and they participated earlier in many minor anomaly studies, they have a long clinical experience in dysmorphology. The examination of minor physical anomalies was done qualitatively (present or absent) without scores being used, but where it was possible, measurements were taken with calipers and tape to improve the objectivity of examination” (9,226.pp).

### Statistical Analysis

“Before the statistical analyses interrater reliability was tested and the kappa coefficient was >0.75 for all items. Statistical analyses were done by applying the Mann - Whitney *U*-test for and the chi-squared test for the comparison of the two groups with each other. Two-sided Fisher's exact tests were used to compare the two groups with each other by body regions, the level of significance chosen was *p* < 0.05. For the analysis of the frequency of each individual minor physical anomaly the two-sided Fisher's exact probability test was used, the level of significance chosen was *p* < 0.05. All the statistical analyses were done by the use of SPSS Version 21” (9,226.pp).

### Results

The comparison of two groups with the Mann-Whitney-*U*-test showed significant differences between them (relatives of bipolar I patients: mean rank: 29.8 vs. normal controls: mean rank: 11.2, *p* < 0.001). The differences of the MPA profiles between the two groups is shown on Table 1. As in our earlier publications (3, 8, 9) we did a dichotomization by establishing two groups: (1) none or only 1 MPA, (2) MPAs more than 1. While in the control group the number of individuals with none or only with 1 MPA was 18 (90%), this in the relative group was 2 (10%), the chi-squared test showed a statistically significant difference (*p* < 0.001). The significantly different between group data of the 2-sided Fisher's exact test comparisons of percentages according to body regions are shown on Table 2. Relatives of bipolar I patients showed a higher frequency of MPAs in the ear, head, mouth and trunk regions compared to normal control subjects. By the differentiation of minor malformations and phenogenetic variants, we have found that phenogenetic variants were more common among the relatives of bipolar I patients compared to the control group (relatives: mean rank: 26.15 vs. controls: mean rank: 14.85, *P* = 0.002), while minor malformations were also more prevalent in the relative group, (relatives: mean rank: 28.43, controls: mean rank: 12.57, *p* < 0.001). Comparing phenogenetic variants by body regions, between the two groups phenogenetic variants in the mouth region were more prevalent (Fisher's exact test, two-sided: 0.003) among the relatives of bipolar I patients. Comparing minor malformations by body regions: ear minor malformations (Fisher's exact test, two-sided: 0.008), trunk minor malformations (Fisher's exact test, two-sided: 0.025) and foot minor malformations (Fisher's exact test, two-sided: 0.047) were more prevalent in the relative group. The results of comparisons of individual MPAs among the two groups are shown on Table 3. Only one minor malformation (sole crease) and one phenogenetic variant (high arched palate) were more prevalent (*p* = 0.047) in the bipolar relatives group compared to the normal control group.

**Table 1:** The number of MPAs in the case of relatives and controls.

	**0 MPA**	**1 MPA**	**2 MPAs**	**3 MPAs**	**4 MPAs**	**5 MPAs**	**6 MPAs**	**7 MPAs**	**8 MPAs**
Controls (no. 20)	11	7	2	0	0	0	0	0	0
Relatives (no. 20)	0	2	3	8	3	2	1	0	1

**Table 2:** Comparison of percentages of body regions in the two goups according to the dichotomization, where the cut point was: (1) at least 1 or more MPAs; (2) 0 MPA.

	**Ear region**	**Head region**	**Mouth region**	**Eye region**	**Trunk region**	**Hand region**	**Foot region**
Controls (no. 20)	0.00%	10.00%	0.00%	0.00%	25.00%	10.00%	10.00%
Relatives (no. 20)	45.00%	45.00%	70.00%	10.00%	65.00%	35.00%	35.00%
Fisher exact test, level of significance	*P* = 0.001 significant	*P* = 0.038 significant	*P* <0.001 significant	*P* = 0.487 ns	*P* = 0.025 significant	*P* = 0.127 ns	*P* = 0.127 ns

*Fisher-exact-test results*.

**Table 3:** Individual analysis of MPAs between the two groups, significant differences.

	**Relatives, number of individuals**	**Controls, number of individuals**	**Ficher exact-test, two-sided**
High arched palate	5	0	*P* = 0.047 significant
Sole crease	5	0	*P* = 0.047 significant

## Discussion

This is the first report in literature on the increased prevalence of minor physical anomalies among the first-degree unaffected relatives of bipolar I patients. Our results on the overrepresentation of the examined anomalies in the relatives of bipolar I patients support the hypothesis, that MPAs may be endophenotypic markers of bipolar I affective disorder. We report here, that minor physical anomalies were more prevalent in the eye, head, mouth and trunk regions among the relatives of bipolar I patients compared to normal controls and the individual analyses showed, that one minor malformation (sole crease) and one phenogenetic variant (high arched palate) were also more prevalent in the relative group compared to the normal control group. First in literature, we report here on the analyses of MPAs among relatives of bipolar I affective patients by the differentiation of minor malformations and phenogenetic variants, and we emphasize that insults resulting aberrant neurodevelopment may appear both during and after the first and second trimester (both phenogenetic variants and minor malformations were more prevalent in the relative group, while one minor malformation and one phenogenetic variant were significantly more common among the relatives of patients during the individual analysis). We see as an important result, that relatives of bipolar I patients showed a higher frequency of MPAs in the eye, head and the mouth regions and one phenogenetic variant (high arched palate) was more prevalent in this group of individuals. Previous findings suggested, that anomalies of the head and the mouth may have more relevance to the hypothetical neurodevelopmental failure in patients with several neuropsychiatric disorders (2, 8, 12, 15, 24–26). In our previous study on the higher prevalence of minor physical anomalies among healthy schizophrenia relatives (9), in harmony with the results of Tikka et al. (26), we reported that MPAs in the craniofacial region were significantly higher in the first-degree relative group than the healthy control group.

### Limitations

A limitation of our study is the relatively smaller sample size, the replication of our findings on bigger samples seems important. Another caveat of the work, that the relative group is heterogeneous, as 11 parents, 3 siblings and 6 children of bipolar I patients were evaluated, 10% of the relatives (2 children) by chance can develop bipolar disorder later.

To conclude: considering the endophenotype concept (9, 17), it should be reminded that although minor physical anomalies are not specific to bipolar affective disorder and are reported in different neurodevelopmental disorders (2, 14, 27), however our first, pioneering results on the more prevalent appearence of these markers among the relatives of patients with bipolar I affective disorder, can suggest these anomalies as endophenotypic traits, emphasizing at aberrant brain development as an important etiological component in bipolar I disorder. Further studies on the MPA profile of healthy relatives of bipolar I patients, correlating with results from structural brain imaging, can clarify the endophenotypic nature of these somatic markers and the nature of genetic and/or enviromental insults on brain maldevelopment.

## Data Availability Statement

The raw data supporting the conclusions of this article will be made available by the authors, without undue reservation.

## Ethics Statement

The studies involving human participants were reviewed and approved by Commitee on Medical Ethics of University of Pécs. The patients/participants provided their written informed consent to participate in this study.

## Author Contributions

TT, GC, and RH: planning and writing the article. TC, TT, GC, VV, AH, MK, and MS: evaluating the research. MH, AT, and SJ: statistical analysis. All authors contributed to the article and approved the submitted version.

## Conflict of Interest

The authors declare that the research was conducted in the absence of any commercial or financial relationships that could be construed as a potential conflict of interest.
